# Operational Transformer: An investigation of epilepsy detection

**DOI:** 10.1007/s10548-026-01214-6

**Published:** 2026-05-12

**Authors:** Omer Bektas, Serkan Kirik, Omer Faruk Goktas, Irem Tasci, Sengul Dogan, Turker Tuncer

**Affiliations:** 1 Department of Pediatrics, Division of Pediatric Neurology, Faculty of Medicine, Ankara University, Ankara, 06100 Turkey; 2 Department of Pediatrics, Division of Pediatric Neurology, Fethi Sekin City Hospital, 23280 Elazig, Turkey; 3 Department of Electronics and Automation, Technical Sciences Vocational School, Ankara Yildirim Beyazit University, Ankara, Turkey; 4 Department of Neurology, Firat University Hospital, Firat University, 23119 Elazig, Turkey; 5 Department of Digital Forensics Engineering, College of Technology, Firat University, 23119 Elazig, Turkey

**Keywords:** Operational Transformer, XFE, Epilepsy detection, EEG signal classification, Connectome Theory, Directed Lobish

## Abstract

Electroencephalography (EEG) signals represent the electrical activities of the brain and have been utilized to assess brain conditions. EEG signals are also crucial for diagnosing epilepsy. However, EEG interpretation is a challenging task. Therefore, new-generation methods should be introduced. The essential goal of this study is to present a new transformer model for multichannel EEG signal classification. A new transformer model has been introduced in this research, termed the Operational Transformer (OpT). To evaluate the classification capability of OpT, a new-generation explainable feature engineering (XFE) framework is presented. The OpT-driven XFE approach comprises four key stages: (i) feature derivation utilizing OpT and a transition table feature extractor to obtain EEG signal attributes, (ii) identification of the most significant features through cumulative weighted iterative neighborhood component analysis (CWINCA), (iii) classification of the selected features via k-nearest neighbors (kNN), and (iv) generation of explainable outputs leveraging the Directed Lobish (DLob)-based interpretation method. These phases were integrated to construct an XFE framework aimed at measuring the efficiency of OpT, which was validated on a publicly available EEG epilepsy dataset. The presented OpT-centric XFE model yielded classification accuracies of 99.99% and 84.74% under 10-fold cross-validation (CV) and leave-one-subject-out (LOSO) CV, respectively. Furthermore, a connectome diagram was generated using DLob for the employed dataset. The computed classification and interpretability results show that the introduced OpT-driven XFE model performs strongly under the reported experimental conditions. The presented XFE model contributes to feature engineering by providing high classification performance and to neuroscience by generating interpretable results utilizing DLob.

## Introduction

Electroencephalography (EEG) is a common neurophysiological instrument used to study electrical brain activity (Asadi et al. 2025; Jackson and Bolger 2014). It is an essential tool for diagnosing a wide range of neurological disorders, most notably epilepsy (R. Sharma and Meena 2024). EEG is a non-invasive procedure in which electrodes are placed on the scalp to record neuronal activity (Manohara et al. 2024). These signals reflect the functional activity of specific brain regions (R. Sharma and Meena 2024).

Epilepsy is a chronic neurological disorder that causes recurrent seizures due to disorganized electrical discharges in the brain (Cresto et al. 2024; Sharma et al. 2024). The disorder has variable clinical expression, and the most common subtypes are focal and generalized epilepsy. Temporal, frontal, and parietal lobe epilepsies are commonly encountered in clinical practice, and each has characteristic EEG features (Gavnholt et al. 2024). Recognizing these patterns is crucial for diagnosis and planning appropriate treatment (Pellinen et al. 2024).

Interpreting epileptic EEG records is particularly challenging because of the diversity in signal morphology and noise (Zhao et al. 2024). Neurologists typically perform time-consuming manual analysis, which may lead to inter-observer variability. Thus, computational models have been proposed to aid in automated epilepsy detection (Brown et al. 2024; Daniyal et al. 2024). These models depend upon extracting informative features from EEG signals followed by their classification via predefined categories.

Recent developments in artificial intelligence and signal processing have given rise to feature engineering methods aimed at improving the classification accuracy of EEG signals (Nafea and Ismail 2024). This process involves statistical transformations, FFT-based analysis, feature selection strategies, and shallow/traditional classifiers to achieve reproducible results for automated epilepsy detection (Edoho et al. 2024). Additionally, explainable artificial intelligence (XAI) techniques are increasingly relevant to clinical applications, as they describe their findings in ways that are consistent with neurologists’ mental models (Ahmad et al. 2024; Elshewey et al. 2024; Rashed-Al-Mahfuz et al. 2021).

This study investigates the use of EEG feature engineering techniques for epilepsy detection and highlights modern strategies for improving classification performance. It also emphasizes the need for interpretability in computational models.

### Literature Review

Some current machine learning-based epilepsy studies in the literature are presented below.

Cao et al. (Cao et al. 2025) introduced an epileptic seizure detection method called hybrid convolutional neural network (CNN)- bidirectional long short-term memory (BiLSTM) model by using feature fusion. In order to ensure diversity of patients used in their study, they utilized EEG data taken from the Bonn, New Delhi, and CHB-MIT datasets. For five-level decomposition of EEG signals, Discrete Wavelet Transform was used, through which both time–frequency and nonlinear features were extracted. For binary classification, the proposed method yielded 100% accuracy, sensitivity, specificity, precision, and F1-score on Bonn and New Delhi datasets. On the Bonn dataset (A-D-E) in a three-class classification taking place on, the accuracy, sensitivity, and precision obtained by the model were 96.19%, 95.08% and 97.49% respectively. Their model’s robustness was further validated using the CHB-MIT dataset, yielding an average accuracy of 98.43%. Gardy et al. (Gardy et al. 2025) developed a wavelet-based CNN detector for the detection of fast ripple in epilepsy from intracerebral EEG (iEEG) macro-contacts and microwires recordings. It used a dataset of 11,000 + fast-ripple events from 38 patients suffering from epilepsy. Training on simulated and real iEEG data, their method achieved a sensitivity of up to 99.95% and a precision of 96.51% in the simulated dataset. Mekruksavanich et al. (Mekruksavanich et al. 2025) suggested a hybrid deep learning classifier that includes CNN, residual bidirectional gated recurrent unit (BiGRU), and convolutional block attention module (CBAM) for epileptic seizure detection. They used the publicly accessible Bonn EEG dataset, comprising five sets (A–E) across varying brain states, including ictal and interictal phases. Their model achieved 99.00% accuracy for binary classification, 96.20% accuracy for three-class classification, 92.00% accuracy for four-class classification, and 89.00% accuracy for five-class classification. Verma et al. (Verma et al. 2025) used continuous wavelet transform and a deep CNN for automated seizure classification and epilepsy detection. They studied the University of Bonn EEG dataset containing various data; the dataset is divided into five subsets: three for normal, interictal, and ictal states, respectively, and two for the standard EEG results. Raw EEG signals were filtered with a band-pass filter (0.53–40 Hz) and transformed to time-frequency scalograms by continuous wavelet transform. With transfer learning, the deep learning model based on AlexNet obtained 100% training accuracy and 94.5% validation accuracy in classifying the segments of EEG into normal, interictal and ictal states. Li et al. (Li et al. 2025a, b) proposed a hybrid deep learning model named CNN-Informer by feeding the model with EEG data from two public datasets, CHB-MIT (983 h and 198 seizures) and SH-SDU (143 h and 103 seizures). DWT and 4-second EEG epoching were applied. Local preserved features were extracted by CNN and Informer was used to capture the long-range dependencies. Their model reached an accuracy of 98.54%, a sensitivity of 99.54% and an event-based false detection rate (FDR) of 0.16/h on CHB-MIT, and an accuracy of 92.78% and an event-based sensitivity of 94.83% on SH-SDU. Liu et al. (Liu et al. 2025) suggested a method called evidential multi-view learning for the purpose of the detection of epileptic seizures. The CHB-MIT dataset of pediatric epilepsy patients’ EEG recordings was used. Using evidential fusion, the evidential multi-view learning model extracted multi-view features (temporal, spectral, temporal-spectral) and fed them into their model separately. They attained accuracy, sensitivity, and specificity of 99.05%, 97.96% and 99.64%, respectively. Shawly et al. (Shawly et al. 2025) developed the multi-attention forward and backward network to predict epileptic seizures from EEG data. In their study, three public datasets were used, including: CHB-MIT (22 patients, 256 Hz), Siena (14 patients, 512 Hz), and ResearchGate (10 patients, 200 Hz) and EEG signals from more than five healthy controls were used. CNN, LSTM, and U-shaped architectures were used for feature extraction and classification. Their model yielded an accuracy of 97.88% and a sensitivity and specificity of 95%–98.01%. Pidvalnyi et al. (Pidvalnyi et al. 2025) presented an EEG-based machine learning approach for the classification of seizures in rats with pilocarpine-induced temporal lobe epilepsy using recorded intracranial EEG (iEEG) data. Support Vector Machine (SVM) was used for classification and PCA for feature selection. Key features that were identified were Hjorth’s parameters and Daubechies discrete wavelet transform coefficients. The SVM model attained a sensitivity of 90% and specificity of 74%. Disli et al. (Dişli et al., 2025) proposed a continuous wavelet transform depthwise CNN on a publicly available 35-channel EEG dataset for seizure diagnosis. After raw EEG signals were transformed into time-frequency images, they were concatenated and used as input to the depthwise CNN model. They reached 95.99% accuracy, 94.27% sensitivity, 97.29% specificity, and 96.34% precision. Hu et al. (Hu et al. 2025) presented an instance-level spatio-temporal memory autoencoder, which performs unsupervised vision-based seizure detection. Their study used video recordings of 15 pediatric epilepsy patients at the Children’s Hospital, Zhejiang University School of Medicine (CHZU). Their study combines YOLOv5 for object detection with a two-stream memory network to capture both spatial and temporal motion features. Their model reached an area under curve of 98.16%. Li et al. (Z. Li et al. 2025a, b) developed a deep learning framework for the detection of epileptic seizure using stereo-electroencephalography (SEEG) signals. The XJSZ dataset included 1,277 h of SEEG recordings collected from eight epilepsy patients with 426 seizure events. On the XJSZ dataset, they attained 99.03% accuracy, 99.34% specificity, and 99.03% sensitivity.

### Literature Gaps

- Numerous models for epilepsy detection and diagnosis have been developed; however, most of them mainly emphasize classification accuracy. As a result, only a limited number of explainable models (XAI-based models) are available for epilepsy detection.
- Deep learning (DL) techniques are widely/forefront used because of their strong classification performance. However, these models often require substantial computational resources and may be computationally expensive in practice.

### Motivation and our Method

In this research, we were inspired by transformers (Vaswani 2017) and operational neural networks (ONNs) (Kiranyaz et al. 2020). Drawing inspiration from these models, we have introduced a new transformer for feature engineering, termed the Operational Transformer (OpT), which is a channel-based transformation model. By using OpT, channel-level relationships are extracted.

One important difference between the proposed OpT and recent transformer-based EEG models is the way feature representation is created. Many transformer-based EEG models first convert the input signal into patches or tokens and then use attention mechanisms to learn global dependencies. Although these strategies can improve representation power, they usually require more complex processing steps and higher computational resources. In contrast, the proposed OpT is a dedicated transformation model for feature extraction. It directly analyzes channel-based relations and produces transformed signals with explicit channel indices. This property is important because it preserves channel identity during feature generation and supports direct mapping to DLob-based interpretation. In addition, the proposed OpT does not require patch embedding, tokenization, or attention computation. Therefore, the presented framework offers a simpler, lighter, and more interpretable alternative for EEG signal analysis. This advantage is especially important in applications where computational efficiency and transparent feature tracing are required.

As seen in the literature gaps section, there are limited explainable artificial intelligence (XAI)-based epilepsy detection studies in the literature. To fill this gap, we have used a transition table feature extractor with OpT to extract features from the channels. Using this methodology, features have been generated from the extracted channels, and these channels have been transformed into DLob symbols to obtain interpretable results. Therefore, we have integrated DLob into the presented OpT-centric XFE model to enhance interpretability.

To address the second gap, we have aimed to present a feature engineering model consisting of four essential phases: (i) feature extraction using OpT and a transition table feature extractor, (ii) feature selection using cumulative weighted iterative neighborhood component analysis (CWINCA) (Cambay et al, 2024), (iii) classification with kNN, and (iv) DLob-centric XAI results generation. All of these phases show overall linear-complexity behavior under the reported configuration. In this aspect, we have introduced a new-generation lightweight EEG signal classification model. In addition, the introduced OpT-centric XFE was tested on a publicly available EEG epilepsy dataset, and the computed classification and XAI results higlight that the presented OpT-centric XFE model is a competitive alternative to deep learning models.

### Innovations and Contributions

*Innovations*:

- In this research, we have presented a new transformation model, termed OpT.
- By utilizing OpT and the transition table feature extractor, a new feature extraction method has been introduced in this study.
- To investigate the interpretability and classification performance of the introduced OpT, a next-generation OpT-centric XFE model is presented.

*Contributions*:

- The recommended OpT-related XFE framework is designed for EEG signal classification. To validate its effectiveness, we evaluated the model on a publicly accessible EEG epilepsy dataset. The developed OpT-centric XFE approach achieved 99.99% and 84.74% classification accuracy through 10-fold cross-validation (CV) and leave-one-subject-out (LOSO) CV, respectively. These results highlight the model’s high classification performance. In this context, the study advances feature engineering by offering a competitive alternative to deep learning-based methodologies.
- By integrating the DLob symbolic language into the presented OpT-centric XFE model, we have obtained a DLob sentence. By analyzing the generated DLob sentence, interpretable results have been obtained. These interpretable results may support neuroscience-oriented interpretation, particularly in epilepsy-related EEG analysis.

## Dataset

This research uses a publicly available EEG epilepsy dataset, termed the Turkish Epilepsy Dataset (Tasci 2023; Tasci et al. 2023). This dataset was collected using an EEG device that has 35 channels and a sampling frequency of 500 Hz. The dataset includes EEG signals from 121 participants. Among them, 50 participants had epileptic EEG signals, while the remaining 71 showed no findings related to epilepsy. The EEG signals were divided into 15-second segments. This dataset contains 4,465 epileptic EEG segments and 5,891 control EEG segments, totaling 10,356 EEG segments from 121 participants.

## Operational Transformer

**Fig. 1:**
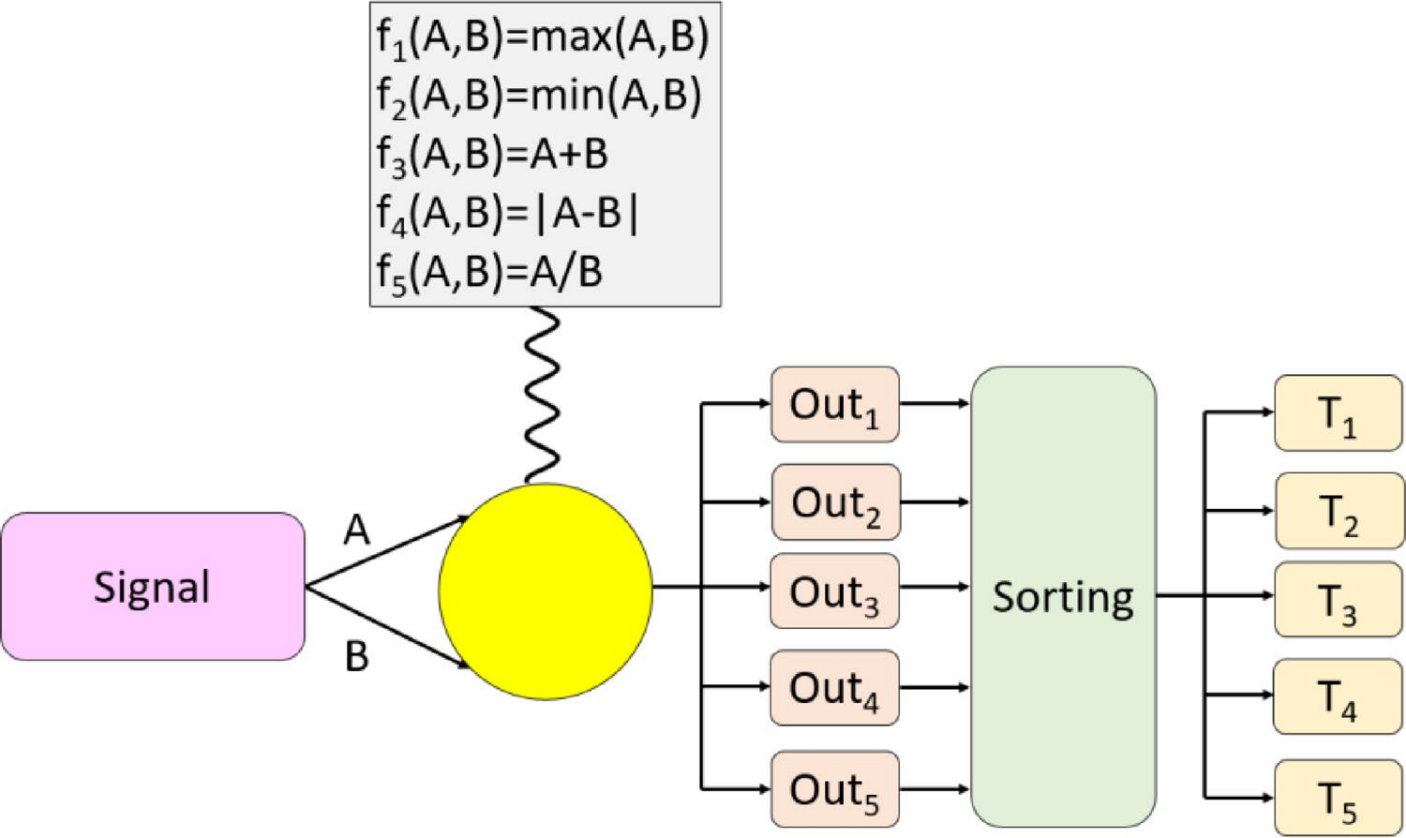
The graphical overview of the presented OpT. Herein, A and B are consecutive channel vectors, Out: output of the statistical moments, T: transformed signals containing the qualified identities

The major innovation of this research is the presented Operational Transformer (OpT). The introduced OpT uses two inputs and produces five outputs by applying five operators. These operators were used to enrich the extracted channel relationships. A sorting operation is then applied to the generated outputs to obtain the channel identities, which were then used to create the transformed signals. The graphical depiction of the presented OpT is shown in Fig. 1 for better clarification.

As seen in Fig. 1, five functions (statistical moments/operators) have been used, resulting in five outputs. The number of functions can be increased to generate more outputs. In this respect, the introduced OpT is a scalable and flexible transformer. The steps of the introduced OpT are as follows:

### S1

Create channel vectors.

1 $$\:A=signal\left(i,1:cn\right),i\in\:\left\{\mathrm{1,2},\dots\:,len-1\right\}\:$$

2 $$\:B=signal\left(i+\mathrm{1,1}:cn\right)$$

where $\:A$ and $\:B$ are consecutive channel vectors, $\:len$: length of the signal and $\:cn$: number of channels of the signal.

### S2

Apply five functions to the extracted channel vectors to create statistical outputs.

3 $$\:ou{t}_{1}\left(j\right)=\mathrm{max}\left(A\left(j\right),B\left(j\right)\right),\:j\in\:\left\{\mathrm{1,2},\dots\:,nc\right\}\:$$

4 $$\:ou{t}_{2}\left(j\right)=\mathrm{min}\left(A\left(j\right),B\left(j\right)\right)$$

5 $$\:ou{t}_{3}\left(j\right)=A\left(j\right)+B\left(j\right)$$

6 $$\:ou{t}_{4}\left(j\right)=\left|A\left(j\right)-B\left(j\right)\right|$$

7 $$\:ou{t}_{5}\left(j\right)=\frac{A\left(j\right)}{B\left(j\right)+\epsilon\:}$$

where $\:out$: the statistical outputs.

### S3

Apply descending sorting to obtain identities and create transformed signals.

8 $$\:i{d}_{x}=argsort\left(ou{t}_{x}\right),\:x\in\:\left\{\mathrm{1,2},\dots\:,5\right\}$$

9 $$\:{T}_{x}\left(a:a+cn-1\right)=idx,\:a\in\:\left\{1,cn+1,\dots\:,len\times\:cn-cn+1\right\}$$

Here, $\:id$: the obtained identities and $\:T$: transformed signal.

### S4

Repeat steps S1 to S3 until the length of the signal is reached, and create transformed signals.

These four steps define the introduced OpT method.

## The OpT-driven XFE model

**Fig. 2:**
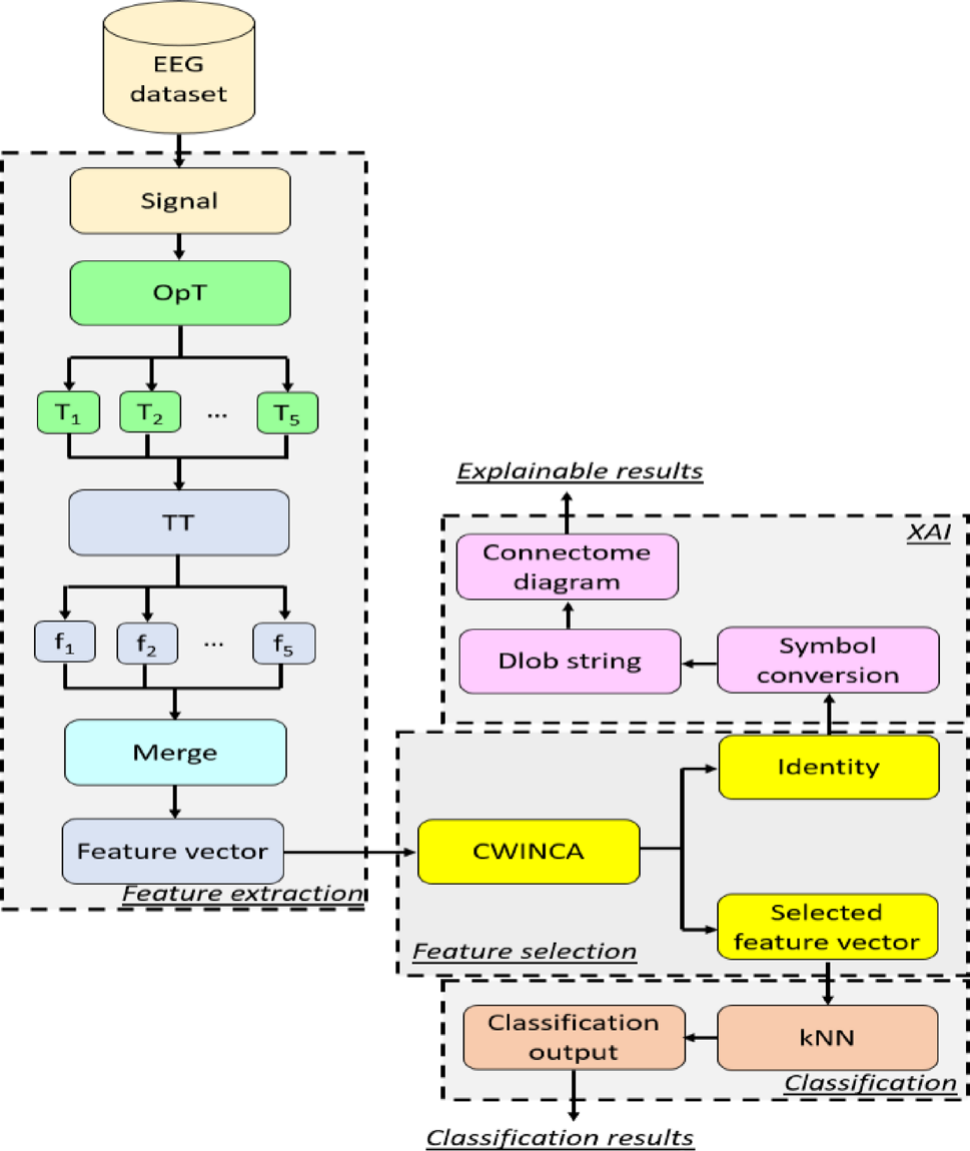
The graphical depiction of the presented OpT-centric XFE model. Herein, T: transformed signal, TT: transition table feature extractor, f: individual feature vector, kNN: k-nearest neighbors

We introduce a new-generation XFE model to investigate the classification capability of the proposed OpT transformer, with the primary objective of evaluating both classification performance and interpretability. Thus, the introduced OpT-centric XFE framework is structured into four key stages: (i) extracting features through OpT and a transition table feature extraction method, (ii) selecting the most relevant features using CWINCA, (iii) performing classification with kNN, and (iv) generating interpretable outcomes via the DLob-based XAI approach. To enhance understanding, Fig. 2 provides a graphical illustration of the designed OpT-driven XFE model.

Figure 2 demonstrates that the presented OpT-centric XFE model generates five transformed signals using OpT, while the transition table feature extractor generates features from these transformed signals, resulting in five feature vectors. By concatenating these feature vectors, the final feature vector is created. In the feature selection phase, the CWINCA feature selector is applied to the merged/final feature vector, selecting the most relevant features and their corresponding identities. The selected feature vector is then used as input for the kNN classifier to produce classification results. The identities of the selected feature vector are also utilized as input for the XAI phase. In this phase, interpretable results are generated. The details of the phases used are explained below.

***Phase 1***: The first phase of the introduced OpT-centric XFE framework is feature extraction. In this phase, two methods are used: (1) the introduced OpT transformer and (2) the transition table feature extractor. First, the introduced OpT transformer is applied to the EEG signals to create transformed signals. Subsequently, features are extracted from the generated transformed signals using the transition table feature extractor. The steps of the introduced OpT and transition table-centric feature extraction method are:

### Step 1

Generate the transformed signals using the introduced OpT.

10 $$\:{T}_{a}=OpT\left(signal\right),a\:\in\:\{\mathrm{1,2},\dots\:,5\}\:$$

where, $\:T$: the created transformed signal and $\:OpT(.)$: the recommended OpT transformer.

### Step 2

Extract features from the transformed signals generated using the transition table feature extractor.

11 $$\:tr{t}_{a}=\left[\begin{array}{ccc}0&\:\cdots\:&\:0\\\:⋮&\:\ddots\:&\:⋮\\\:0&\:\cdots\:&\:0\end{array}\right]\:$$

12 $$\:tr{t}_{a}\left({T}_{a}\left(h\right),{T}_{a}\left(h+1\right)\right)+=1$$

13 $$\begin{aligned}{f}_{a}\left(u\right)&=tr{t}_{a}\left(q,w\right),\:\left(q,w\right)\in\:\left\{\mathrm{1,2},\dots\:,cn\right\},\\&\quad\:u\in\:\left\{\mathrm{1,2},\dots\:,c{n}^{2}\right\}\end{aligned}$$

where $\:trt$: transition table and $\:f$: the created individual feature vector.

### Step 3

Merge the generated individual feature vectors to create the final feature vector.

14 $$\:F\left(u+c{n}^{2}\left(a-1\right)\right)={f}_{a}\left(u\right)$$

Herein, $\:F$: final feature vector with a length of $\:5c{n}^{2}$.

### Step 4

Repeat Steps 1–3 until the desired number of EEG signals is achieved and create the final feature matrix.

By using these four steps (S1-S4), features are extracted. In this study, 6,125 (= 35 × 35 × 5) features were extracted from each EEG signal since the EEG signal dataset used has 35 channels.

### Phase 2

For feature selection, a self-organized and iterative feature selector has been utilized, specifically the CWINCA (Cambay et al., 2024; Tuncer et al. 2024) feature selector. In CWINCA, the iteration range is established through cumulative weights and two predefined threshold values. In this study, we set 0.5 and 0.99 as threshold values to determine the starting and stopping points of the iteration process. By executing this loop, an iterative feature selection process was carried out, and the classification performance of the chosen features was assessed using the kNN classifier (Maillo et al. 2017). Based on the evaluated classification accuracies, the most effective feature vector was identified. Furthermore, the identities of the selected features were utilized as input for the DLob-based XAI result generation step (Phase 4).

### Step 5

Produce the selected feature vector and the identities of the selected features by using the CWINCA feature selector.

15 $$\:[ind,SX]=CW(X,y)\:$$

where $\:ind$: the indices/identities of the selected feature vector, $\:SX$: the chosen features, $\:CW(.)$: the utilized CWINCA feature selector,$\:\:X$: the generated feature matrix and $\:y$: the actual label.

### Phase 3

To obtain classification results, a straightforward classifier was utilized to highlight the strong discriminative capability of the extracted features. Consequently, the kNN classifier was chosen for generating classification outcomes. In this work, two validation protocols were used. The 10-fold cross-validation was performed at the segment level on the pre-segmented 15-second EEG samples in the Turkish Epilepsy Dataset. Since the dataset contains multiple segments from the same participants, this protocol is not subject-independent and may place highly similar samples from the same participant in both training and test folds. Therefore, the 10-fold CV result should be interpreted as a segment-level within-dataset evaluation. In addition, 10-fold CV is one of the most commonly used validation techniques in the machine learning literature. Therefore, it was also included in this study. To provide a stricter subject-independent evaluation, LOSO cross-validation was also applied. In LOSO CV, all segments from one participant were used for testing, and the remaining participants were used for training. For this reason, LOSO CV was considered the main indicator of cross-subject generalization in this study.

### Step 6

Classify the selected features using the kNN classifier.

16 $$\:ou{t}^{c}=kNN(SX,y)\:$$

Herein, $\:ou{t}^{c}$: the generated classification outcome and $\:kNN(.):$ kNN classifier.

By utilizing the generated classification outcome, the classification results of the presented OpT-centric XFE model have been computed.

### Phase 4

The DLob (Tuncer et al. 2024) symbolic language is utilized in this phase to generate interpretable results. The extracted features contain channel information. Therefore, by extracting these channel numbers from the selected features’ identities, the corresponding DLob symbols have been obtained. Using the extracted DLob symbols, a DLob string has been created. Subsequently, explainable results were derived by analyzing the generated DLob string/sentence. To obtain these results, we extracted the histogram, transition table (for creating the connectome diagram), and Shannon entropy. The steps of this phase are given as follows.

### Step 7

Create the DLob string by using the identities of the selected features.

17 $$\begin{aligned}\:id\left(v\right)&=\lfloor\frac{ind\left(m\right)-1}{nc}\rfloor\:\left(mod\:nc\right)+1,\\&\quad m\in\:\left\{\mathrm{1,2},\dots\:,nsf\right\},\:v\in\:\left\{\mathrm{1,3},\dots\:,2nsf-1\right\}\:\end{aligned}$$

18 $$\:id\left(v+1\right)=ind\left(m\right)-1\:\left(mod\:nc\right)+1$$

19 $$\begin{aligned}\:LUT&=\{FL,\:FR,\:FL,FR,\:Fz,\:CL,\:CR,Cz,PL,\\&\quad\:PR,\:Pz,\:OL,OR,\:FL,\:FR,TL,TR,TL,TR,FL,FL,\\&\quad TL,TL,FL,FL,CL,\:PL,\:PR,FR,CR,PR,FR,FR,TR,TR\}\end{aligned}$$

20 $$\:DS\left(p\right)=LUT\left(id\left(p\right)\right),\:p\in\:\left\{\mathrm{1,2},\dots\:,2nsf\right\}$$

where $\:id$: the channel numbers, $\:nsf$: number of the selected feature vector, $\:LUT$: the used look-up-table for generating DLob symbols from channel numbers and $\:DS$: the created DLob symbol.

As seen in this step, we have used 13 DLob symbols based on the EEG signal collection device. These DLob symbols are FL, FR, Fz, TL, TR, PL, PR, Pz, OL, OR, CL, CR, and Cz. The meanings of these symbols are given below:

FL: Facilitates planning, logical reasoning, and problem-solving.

FR: Governs creativity, emotional modulation, and intuition.

Fz: Regulates attention, focus, and executive functions.

TL: Handles language comprehension, memory, and auditory processing.

TR: Supports emotional processing, memory retention, and non-verbal auditory perception.

OL: Analyzes visual elements like shapes and colors.

OR: Specializes in visual-spatial recognition and processing.

PL: Integrates sensory inputs and manages spatial awareness.

PR: Interprets sensory stimuli and spatial orientation.

Pz: Synthesizes sensory data and enhances spatial cognition.

CL: Coordinates motor control and bodily movements.

CR: Assists in motor regulation and sensory-motor integration.

Cz: Essential for motor planning and execution.

By using the meanings given above, explanations can be extracted from the generated DLob string.

### Step 8

Analyze the produced DLob symbols. In this study, histogram extraction, information entropy computation, and transition table calculation were used to obtain interpretable (XAI) results.

21 $$\:histo\left(n\right)=0,\:n\in\:\{\mathrm{1,2},\dots\:,13\}$$

22 $$\:histo\left(id\left(p\right)\right)+=1$$

23 $$\:cd=\left[\begin{array}{ccc}0&\:\cdots\:&\:0\\\:\vdots&\:\ddots\:&\:\vdots\\\:0&\:\cdots\:&\:0\end{array}\right]$$

24 $$\:cd\left(id\left(r\right),id\left(r+1\right)\right)+=1,\:r\in\:\left\{\mathrm{1,2},\dots\:,2nsf-1\right\}$$

25 $$\:pr\left(n\right)=\frac{histo\left(n\right)}{\sum\:_{i=1}^{n}histo\left(n\right)}$$

26 $$\:ent=-\:\sum\:_{n=1}^{13}pr\times\:\mathrm{l}\mathrm{o}\mathrm{g}\left(pr\right)$$

Herein, $\:histo$: histogram of the DLob symbols, $\:cd$: connectome diagram matrix, $\:pr$: probability of the DLob symbol and $\:ent$: information entropy of the generated DLob string.

## Experimental Results

The introduced OpT-driven XFE model was tested on the Turkish Epilepsy Dataset. First, we downloaded this dataset from the Kaggle website. Our model was implemented on a simple-configured laptop using MATLAB R2024a. By utilizing MATLAB, the functions of the presented XFE model were created and stored as m files. The presented OpT-centric XFE model is lightweight because it shows overall linear-complexity behavior under the reported configuration and the framework is also flexible because different machine learning models can be integrated into it. The time complexity analysis and parameters of this model are demonstrated in Table 1.

**Table 1:** Time complexity (Big O notation) analysis and the parameters used for the presented OpT-driven XFE model

Phase	Method	Parameters	Time complexity
Feature extraction	OpT	The used statistical moments: Minimum, maximum, addition, distance and division. Number of the generated transformed signals: 5.	$\:O\left(L\right)$
	Transition table feature extractor	Size of the transition table:$\:c{n}^{2}$.	$\:O\left(L\right)$
	Feature merging	Concatenation	$\:O\left(L\right)$
Feature selection	CWINCA	Threshold values: 0.5, 0.99, Start value: 41, Stop value: 163, Number of the selected feature vectors in the iteration: 83, The length of the final selected feature: 72.	O(N+RK+G)
Classification	kNN	k:1, Distance: L1-norm, Weight: Equal, Validation: 10-fold / LOSO CVs	$\:O\left(K\right)$
XAI	DLob-based XAI generation	Number of the DLob symbols used: 13, Number of the DLob symbols in the generated DLob string: 144, The used statistical analysis methods: Histogram extraction, information entropy computation and transition table extraction	$\:O\left(S\right)$

** Herein,$\:L$: length of the signal,$\:N$: time complexity coefficient of the NCA feature selector,$\:R$: range of the iteration,$\:K$: time complexity coefficient of the kNN classifier,$\:G$: time burden coefficient of the greedy algorithm,$\:S$: length of the selected features

According to Table 1, the time burden of the introduced OpT-related XFE model is computed as $\:O(L+N+RK+G+S)$. The computed time burden result shows the overall linear-complexity behavior of the introduced OpT-driven XFE model. Moreover, we implemented the presented model using the given parameters.

From a practical viewpoint, the computational cost of the proposed model is dominated by the feature selection phase. The OpT transformation, transition table feature extraction, feature merging, and DLob-based interpretability stages all operate with linear-complexity behavior and therefore introduce a limited runtime burden. The CWINCA stage is relatively more demanding because it performs iterative evaluation and greedy selection. However, this step produces a compact final feature vector, which also reduces the burden of the classification stage. In addition, the proposed OpT-driven XFE framework does not include parameter-heavy deep training, backpropagation, patch embedding, or attention-based computation. This makes the method simpler and lighter in practical use. Since the model was implemented in MATLAB on a simple-configured laptop, the presented framework can be considered suitable for standard computing environments.

This model generates two outputs: (i) classification and (ii) explainable outputs. The results obtained from these outputs are presented below.

### Classification Results

To assess classification performance, the kNN classifier was applied using two validation approaches: (i) 10-fold CV and (ii) LOSO CV. These validation methods were used to improve the robustness and reliability of the classification results.

For performance evaluation, four key metrics were considered: (1) classification accuracy, (2) sensitivity, (3) specificity, and (4) geometric mean. Additionally, the confusion matrices used to compute these classification metrics are presented below.

**Fig. 3:**
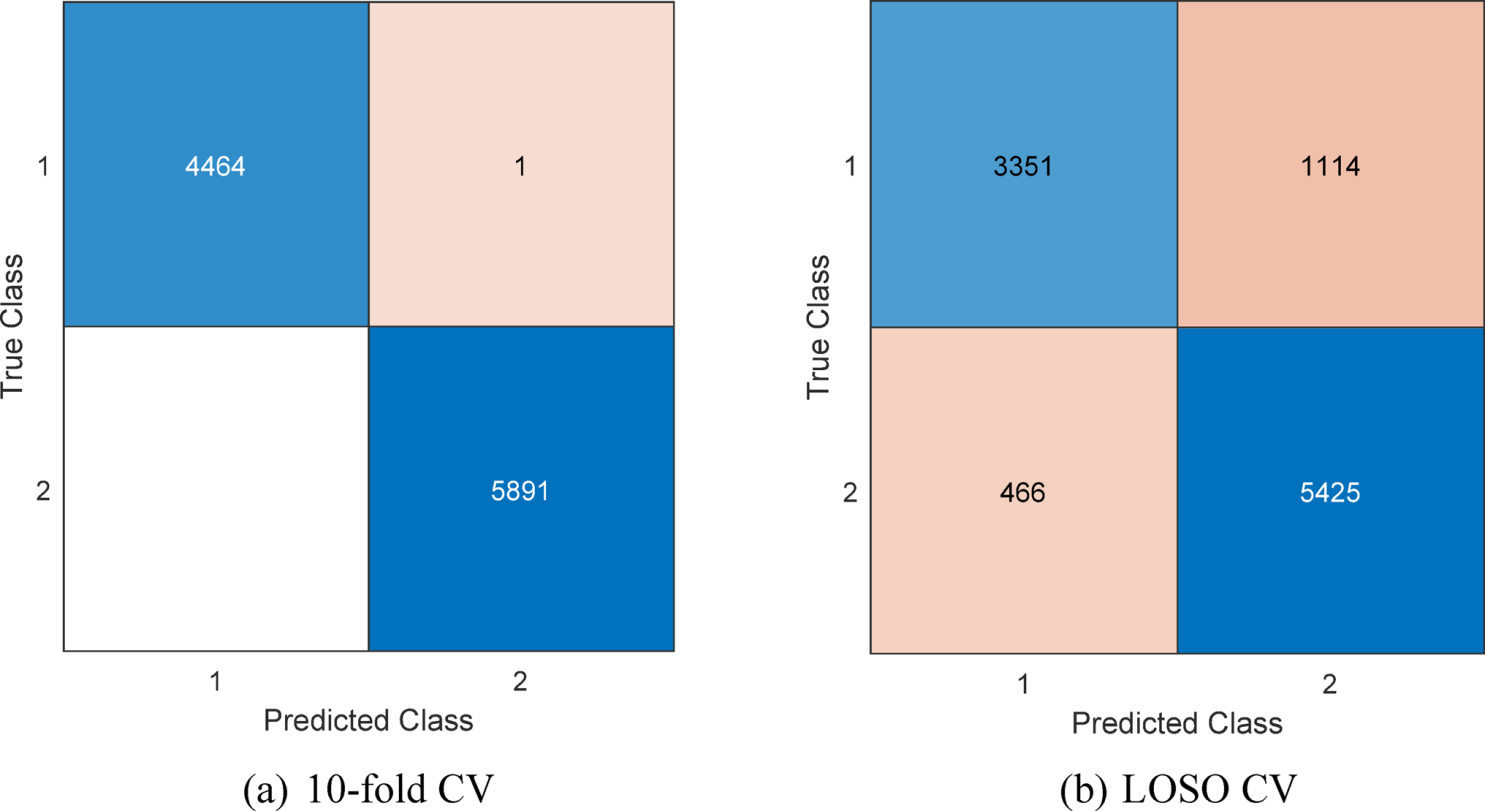
The confusion matrices of the presented OpT-driven XFE model. Herein, 1: Epilepsy, 2: Control

Based on the computed confusion matrices (see Fig. 3), the classification results are listed in Table 2.

**Table 2:** The classification performances (%) of the presented OpT-centric XFE model

Metric	Validation	
	10-fold CV	LOSO CV
Classification accuracy	99.99	84.74
Sensitivity	99.98	75.05
Specificity	100	92.09
Geometric mean	99.99	81.46

Table 2 highlights that the introduced OpT-driven XFE model achieved classification accuracies of 99.99% and 84.74% using 10-fold CV and LOSO CV, respectively.

The difference between the 10-fold CV and LOSO CV results shows that within-dataset classification is easier than subject-independent classification. In this study, LOSO CV should be considered the more reliable validation method because it tests the model on a completely unseen participant. Therefore, LOSO CV gives a more realistic measure of cross-subject generalization. However, LOSO CV also has some practical disadvantages. EEG signals may vary across participants because of differences in head size, head shape, cap placement, and electrode positioning. Therefore, the same channel labels may not correspond to exactly the same anatomical regions in all participants. This increases inter-subject variability and makes subject-independent classification more difficult. As a result, the LOSO CV performance is lower than the 10-fold CV performance. In contrast, the 10-fold CV result reflects a less strict evaluation and may appear more optimistic.

### Explainable Results

The second output of the presented OpT-related XFE model includes explainable results. To obtain these results, a DLob sentence has been generated. The generated DLob sentence, which contains 144 DLob symbols (288 letters), is provided below.

TRCRTRFzTRFLTRFRTRCRTRCLTRPLTRCzTRTRTRFLTROLTRTLTRPRTROLTRTRTRPRTRTLTRCRTRFLTRTRTRPRTRPzTRPLTRPLTRFRTRFRTRPLTRCLTRFRTROLTRPRTRCLTRTRTRFLTRFRTRFRTRFRTRPRTRTLTRPLTRPRTRFRTRCLTRPRTRPLTRTRTRFLTRORTRFLTRFRTRTRTRFRTRFLTRTRTRFLTRFLTRTLTRFLTRCLTRTLTRPRTRFLTRPLTRFLTRFLTRPzTRTLTRPRTRPRTRCRTRTRTRPR.

By analyzing this DLob string, explainable findings were obtained. The histogram and connectome diagram of this DLob string are depicted below.

**Fig. 4:**
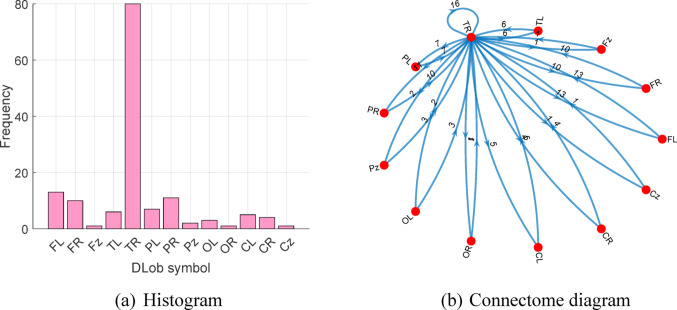
The explainable results

By using this DLob string, explainable results were obtained. The histogram and connectome diagram of this DLob string are shown in Fig. 4. According to the histogram, the most frequent DLob symbol is TR. This result indicates that temporal-region-related symbolic features were selected more often than the other symbolic features in the proposed model. In addition, frontal and parietal symbols such as FL, FR, PL, and PR are also observed. This finding shows that the selected discriminative features are not limited to a single region. It also suggests that the model uses a distributed spatial pattern.

The connectome diagram also supports this interpretation. In the connectome representation, the TR symbol has the strongest and most frequent connections with the other symbols. This result indicates that TR is the main symbolic center in the generated DLob structure. However, this diagram should be interpreted as a relationship map of the selected symbolic features. It should not be considered a direct clinical connectivity map or a direct seizure localization map.

The Shannon entropy of the generated DLob string was calculated as 2.4015. The maximum possible entropy for 13 DLob symbols is 3.7004 (= log_2_13). Based on these two values, the complexity ratio was calculated as 64.90%. This value indicates that the generated symbolic pattern has a moderate level of structural regularity. It shows that the selected symbolic sequence is not fully random. However, this result does not directly confirm a fixed clinical pattern or a specific neurophysiological mechanism.

In the present study, the DLob-based interpretability outputs were not quantitatively validated with external clinical labels, expert annotations, or anatomical ground truth. Therefore, the DLob-based findings should be considered model-based and hypothesis-generating explanations. They provide an interpretable summary of how the selected discriminative features are distributed across symbolic brain regions. These findings may support future clinical interpretation; however, further validation is still needed.

## Discussions

The introduced OpT-centric XFE model showed high and reliable classification performance, with 99.99% accuracy under 10-fold CV and 84.74% accuracy under LOSO CV. In this respect, the developed model exhibits high classification effectiveness. Additionally, all other models focused solely on classification accuracy, whereas our model also generated XAI results using DLob. Our model advances both feature engineering (as a competitive alternative to deep learning) and neuroscience (via the interpretable DLob string, where each symbol maps to a cortical area).

According to the XAI results, the most activated DLob symbol is TR (right temporal lobe) and this result highlights that temporal-region-related symbolic features were selected more often by the model and our model detects the abnormality in this cortical area. Moreover, frontal and parietal epilepsy-related EEG patterns are also present. By analyzing the histogram of the DLob string, model-based spatial patterns related to the selected discriminative features can be observed.

### Neurological Interpretation

The connectome findings (DLob results) provide supportive information about how the model forms its decision. In the generated DLob structure, the TR symbol is the dominant node and it has the strongest connections with the other symbols. This finding suggests that temporal-region-related symbolic features contributed more strongly to the final model output than the other symbolic regions. This result is clinically reasonable because the evaluated dataset contains many participants with temporal epilepsy. Therefore, the prominence of TR may be related to the distribution of epilepsy types in the used dataset.

Another important finding is the presence of frontal and parietal symbols such as FL, FR, PL, and PR in both the histogram and the connectome structure. This result suggests that the model does not rely only on temporal-region-related symbolic information. It also uses additional spatial information from other symbolic regions. This may indicate that the discriminative EEG pattern is not limited to a single symbolic region. Instead, the model appears to use a more distributed spatial decision pattern.

The central role of TR in the connectome diagram may also indicate that temporal-region-related features act as the main reference point in the selected symbolic structure, while frontal and parietal features provide supportive information. In this respect, the connectome diagram may help explain how the proposed model organizes region-related symbolic features during the classification process. However, this interpretation should remain at the model level. The presented connectome should not be considered a direct clinical connectivity map. It should not be interpreted as a definite seizure propagation map or a direct seizure focus localization result.

The obtained connectome findings show that our model highlights a temporal-dominant but spatially distributed symbolic pattern in epilepsy detection. This interpretation is consistent with the composition of the evaluated dataset and may provide supportive clinical meaning. However, further validation with expert annotations, seizure focus labels, and anatomical references is still needed before stronger clinical conclusions can be made.

### Comparative Results

One of our key motivations is to propose a competitive alternative to deep learning models. Therefore, we have compared the obtained classification results with other state-of-the-art (SOTA) models, and the comparative results are presented in Table 3.

**Table 3:** The comparative results

Model	Accuracy (%)	XAI
Hypercube Pattern (Tasci et al. 2023)	10-fold CV: 87.78 LOSO CV: 79.95	-
Xception (Chollet 2017)	10-fold CV: 87.42	-
VGG16 (Simonyan and Zisserman 2014)	10-fold CV: 91.13	-
Transformer (Vaswani 2017)	10-fold CV: 85	
MobileNet (Sandler et al. 2018)	10-fold CV: 91.66	-
CWT-based DCNN (Dişli et al., 2025)	10-fold CV: 95.99	-
Archimedean Spiral and Swin Transformer	10-fold CV: 97.98	-
OpT-centric XFE	10-fold CV: 99.99 LOSO CV: 84.74	DLob-based XFE

Table 3 depicts that the presented model achieved the highest classification performance.

### Ablation Study

The ablation results are presented in this section. In this research, a simple classifier, kNN, was used. We selected the kNN classifier using MATLAB’s Classification Learner Toolbox, which includes various classifiers. Among these, kNN demonstrated the highest classification accuracy. Therefore, we used kNN to generate classification results.

**Fig. 5:**
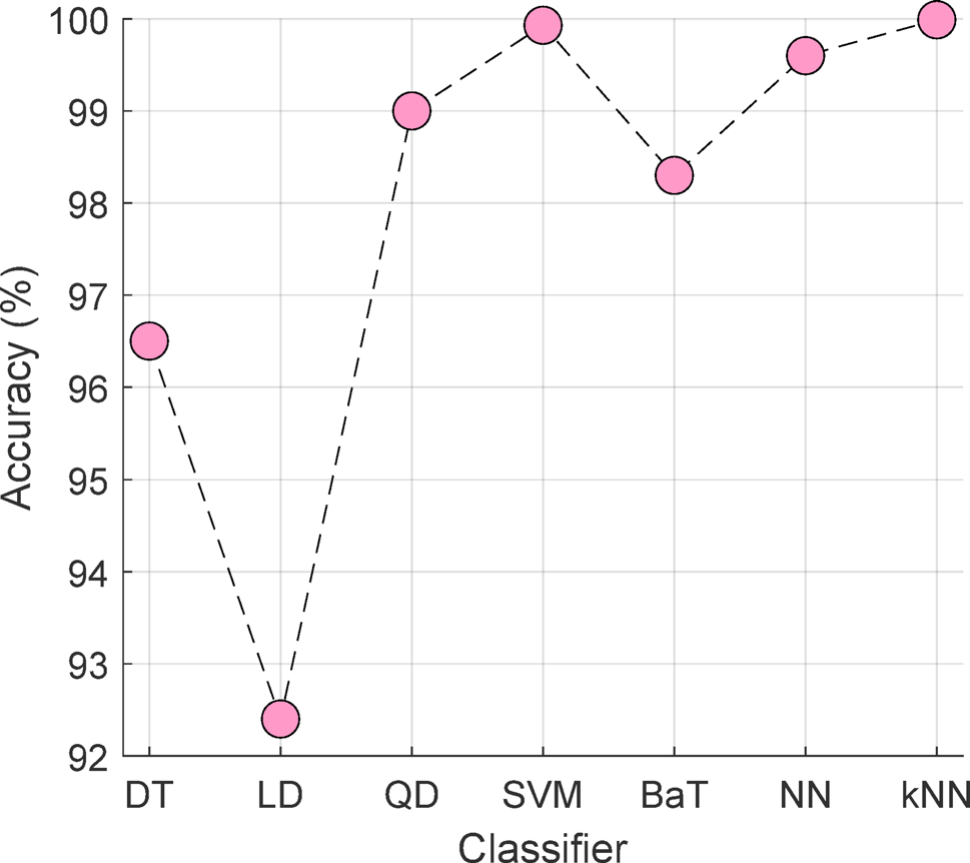
The classification results of the used classifiers

To evaluate the relative performance of the kNN classifier, we compared its performance with Decision Tree (DT) (Safavian and Landgrebe 1991), Linear Discriminant (LD) (Zhang et al. 2007), Quadratic Discriminant (QD) (Bhattacharyya et al. 2010), Support Vector Machine (SVM) (Vapnik 1998), Bagged Tree (BaT) (Hothorn and Lausen 2003), Neural Network (NN) (Denoeux 2000), and kNN (Maillo et al. 2017) itself. The obtained classification results using 10-fold CV are presented in Fig. 5.

Per Fig. 5, the kNN classifier achieved 99.99%, while Cubic SVM attained 99.95% classification accuracy on the dataset. The worst-performing classifier was LD (Linear Discriminant), which achieved 92.57% classification accuracy using the selected feature vector.

**Fig. 6:**
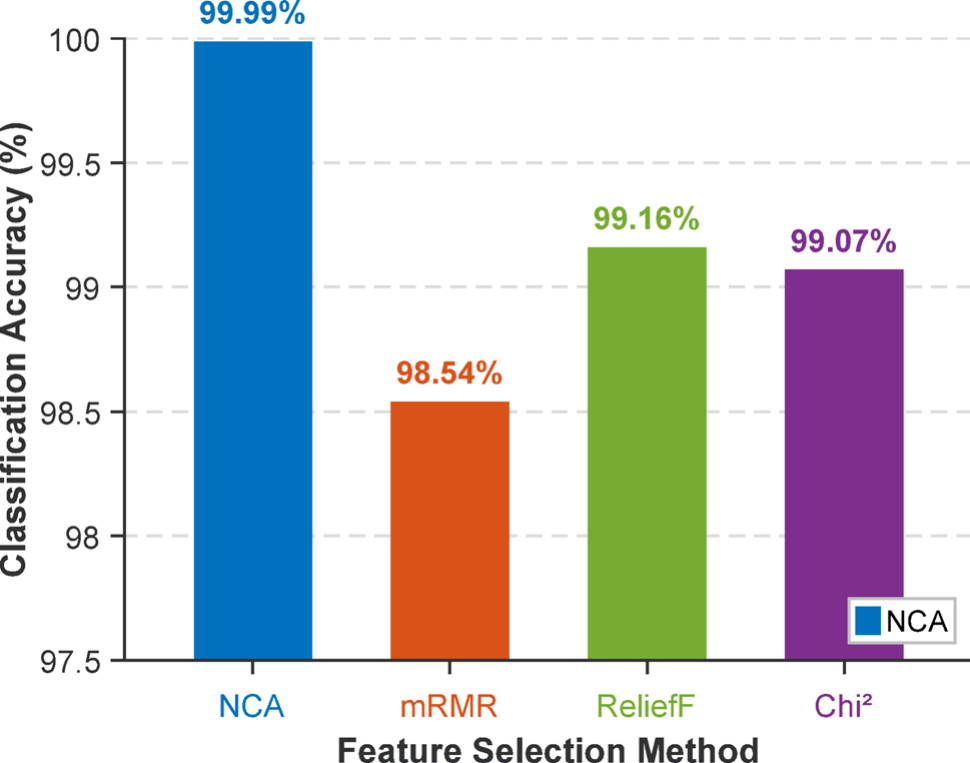
Comparison of feature selector accuracies with 10-fold CV

A brief ablation study was conducted to evaluate the effect of the feature selection stage on the classification performance of the proposed model. In this study, the main structure of the presented OpT-driven XFE pipeline was kept the same. The OpT-based feature extraction, transition table generation, feature merging, and classification stages were not changed. Only the feature selection method was changed. For this purpose, four feature selection methods were tested: NCA (Goldberger et al. 2004), mRMR (Peng et al. 2005), ReliefF (Kononenko 1994), and Chi2 (H. Liu and Setiono 1995). The computed ablation results for feature selectors are illustrated in Fig. 6.

Figure 6 highlights that classification performance changes according to the selected feature selector. The NCA-based setting achieved the highest accuracy with 99.99%. The mRMR-based setting achieved 98.54% accuracy. The ReliefF-based setting achieved 99.16% accuracy. The Chi2-based setting achieved 99.07% accuracy. These results show that the feature selection stage directly affects the final classification performance of the proposed framework.

Among the tested methods, NCA produced the best result. This finding indicates that NCA selected the most discriminative features for the studied dataset. Although mRMR, ReliefF, and Chi2 also achieved high accuracy, their results were lower than the NCA-based setting. Therefore, the ablation study highlights that the proposed model remains effective with different feature selection methods, while the best performance was obtained with NCA. This result also confirms that the feature selection stage is an important part of the introduced OpT-driven XFE model.

### Tests on the Additional Datasets

To further evaluate the general classification capability of the introduced, OpT-driven XFE framework, we tested it on two additional datasets: (i) EEG stress detection dataset and (ii) EEG artifact classification dataset. The classification results obtained using the OpT-centric XFE framework for these datasets are summarized in Table 4.

For the EEG stress detection dataset, the model achieved 76.33% accuracy with LOSO CV and 99.95% accuracy using 10-fold CV. Additionally, for the EEG artifact classification dataset, it reached 87.95% accuracy with 10-fold CV across eight classes.

To further validate the model’s effectiveness, its performance was compared with state-of-the-art (SOTA) models, and the comparative results for these additional datasets are presented in Table 4.

**Table 4:** The comparative results for EEG stress and EEG artifact classification datasets

EEG Artifact Classification Dataset		
Model	Validation	Accuracy (%)
Directed Lobish (Tuncer et al. 2024)	10-fold CV	77.58
OpT-centric XFE	10-fold CV	87.95%
EEG Stress Detection Dataset		
ChMinMaxPat (Bektas et al. 2024)	10-fold CV	92.86
ChMinMaxPat (Bektas et al. 2024)	LOSO CV	73.30
QuadTPat (Cambay et al. 2024a, b)	10-fold CV	92.95
QuadTPat (Cambay et al. 2024a, b)	LOSO CV	73.63
OpT-centric XFE	10-fold CV	99.95
OpT-centric XFE	LOSO CV	76.33

Table 4 lists that the introduced OpT-driven XFE framework achieved higher classification performance compared to other SOTA models. The model showed strong classification performance on the evaluated dataset, while broader generalization should be tested in future studies.

### Relevance to EEG-based BCI Applications

Although the essential objective of this research is epilepsy detection, the proposed OpT-centric XFE framework may also be discussed for EEG-based BCI applications. BCI systems generally aim to decode EEG signals and convert them into predefined commands or classes (Edelman et al. 2024). Therefore, many BCI tasks can also be considered EEG signal classification problems.

The proposed framework has several properties that may be useful for BCI studies. First, OpT-centric XFE is a multichannel EEG classification model. It can extract channel-based relationships from EEG signals. This property may help represent spatial information in BCI-related EEG data. Second, the model is lightweight because it does not require deep training, patch embedding, or attention computation. This structure may be useful for BCI systems, where fast and low-cost processing is important. Third, the model can generate explainable outputs through DLob. This may help researchers understand which channel-related symbolic features contribute to the classification result.

However, the presented study does not directly validate the model for BCI applications. The experiments were performed on offline EEG data for epilepsy detection. In contrast, practical BCI systems usually require task-specific EEG datasets, short decision windows, real-time processing, and online evaluation. BCI systems also need robustness against subject variability, session variability, and non-stationary EEG signals. Therefore, the current results should be interpreted as a methodological potential, not as direct evidence of BCI performance.

The similarity between epilepsy detection and BCI decoding at the methodological level. Both problems require the extraction of meaningful information from EEG signals and the classification of this information into predefined labels. In epilepsy detection, these labels are generally clinical classes. In BCI systems, these labels may represent motor imagery tasks, attention states, emotional states, or user commands. Therefore, the channel-based feature extraction ability of OpT may also be useful for BCI-oriented EEG decoding. In addition, the DLob-based explanation stage may provide a transparent view of the selected channel-related patterns. This property can be useful in BCI studies because model decisions should be both fast and interpretable. However, this possible use requires separate validation on BCI-specific datasets.

In future studies, the introduced OpT-centric XFE can be tested on motor imagery, attention, emotion, or other BCI-related EEG datasets. The model can also be adapted to shorter EEG windows and online classification settings. These future tests may show whether the proposed framework can be used as a lightweight and explainable EEG decoding module for non-invasive BCI systems.

### Highlights

#### Findings

The introduced OpT applies a channel-based transformation strategy to define relationships among EEG channels. This strategy allows inter-channel information to be represented in feature space. CWINCA was used as a self-organized feature selection method to improve classification performance and reduce redundant information. OpT-centric XFE extracted 6,125 features in total, and 72 informative features were selected by CWINCA. These features were then classified with kNN. The model achieved 99.99% accuracy with 10-fold cross-validation and 84.74% accuracy with LOSO cross-validation. These results indicate that OpT has strong classification ability in both sample-based and subject-independent evaluations. OpT also provides explainable outputs with DLob. Each selected feature contains two-channel information. For this reason, the generated DLob sentence included 144 symbols, calculated as 72 × 2. This sentence was examined to obtain cortical-level information, and a cortical connectome diagram was produced. In this diagram, TR was observed as the central node. DLob analysis showed that temporal-region-related symbols were selected more often than other regional symbols. This result indicates a temporal-dominant symbolic pattern in the evaluated dataset. Frontal- and parietal-region-related symbols were also observed. This finding shows that OpT does not rely on only one cortical area. Instead, it uses a distributed symbolic pattern across different brain regions. The complexity ratio of the DLob sentence was 64.90%. This value indicates moderate structural regularity and shows that the symbolic structure is not random. In this respect, the result supports the interpretability capacity of OpT-centric XFE. Two additional EEG datasets were also used to evaluate the general classification ability of OpT-centric XFE. This analysis further supports the robustness and broader applicability of the proposed feature engineering approach.

#### Advantages

Recent transformer-based EEG models generally use patch embedding, token generation, and attention mechanisms. These structures are powerful. However, they usually have higher computational cost, higher memory demand, and more complex architectures. OpT has a different design because it is not a full end-to-end deep transformer model. Instead, it is a transformation-based feature extraction model. OpT directly transforms channel pairs and channel vectors to generate channel-aware transformed signals and channel indices. This structure makes the model simpler, lighter, and easier to interpret. It also provides a better fit with the DLob stage, since extracted features preserve explicit channel identity. The proposed OpT-centric XFE model achieved 99.99% classification accuracy with 10-fold cross-validation and 84.74% classification accuracy with LOSO cross-validation, respectively. The use of both validation schemes provides a broader evaluation under segment-level and subject-independent conditions. Comparative results also show that the OpT-driven XFE framework outperformed deep learning architectures on the evaluated dataset. In addition, tests on two supplementary EEG datasets supported the adaptability and general applicability of the model. Therefore, this study presents a lightweight, accurate, and explainable framework for epilepsy detection.

#### Limitation

The LOSO CV results are lower than the 10-fold CV results because subject-independent evaluation is more challenging.

#### Future directions

Future studies may develop new-generation OpT-like models to improve classification performance, especially under LOSO cross-validation. This direction is important because LOSO validation provides a more realistic subject-independent evaluation. Other biomedical signal datasets may also be used to test the classification ability of the proposed OpT-centric XFE framework on different modalities. In addition, OpT may be integrated into deep learning architectures to examine its contribution in hybrid learning models. Another future direction is related to DLob outputs. Custom language models may be developed to improve the readability and interpretation of DLob-based symbolic results. These future studies may further increase the performance, applicability, and interpretability of the recommended OpT-centric XFE.

#### Potential applications

New-generation personalized medicine applications may be developed to monitor EEG signals of epilepsy patients. In this direction, OpT-centric XFE can support patient-specific analysis and long-term follow-up. Stress and depression monitoring applications may also be implemented with this model to detect possible anxiety-related sources from EEG signals. In addition, OpT-centric XFE can be used in long-term medication monitoring to generate cortical connectome diagrams and explainable insights. This property may help clinicians observe brain-related changes during treatment. The proposed model may also be applied to cardiac signals for heart condition monitoring. Through the extraction and analysis of cardiac signal features, OpT-centric XFE may support the detection of arrhythmia, myocardial infarction, and other cardiovascular abnormalities. DLob-based interpretability may also improve clinical transparency and help cardiologists evaluate model outputs more clearly. Furthermore, the introduced OpT-centric XFE can be applied to other biomedical signals to obtain interpretable and clinically useful insights.

## Conclusions

This work recommended the OpT-centric XFE model for multichannel EEG signal classification. The proposed framework was used for epilepsy detection on a publicly available EEG dataset. The model includes OpT-based channel transformation, transition table feature extraction, CWINCA-based feature selection, kNN classification, and DLob-based explainability. This structure provides both classification results and model-based interpretable outputs.

The experimental results showed that OpT-centric XFE achieved 99.99% accuracy with 10-fold cross-validation and 84.74% accuracy with LOSO cross-validation. The 10-fold CV result shows strong segment-level performance. However, the LOSO CV result is more important for subject-independent evaluation because it tests the model on unseen participants. The lower LOSO CV performance shows that cross-subject EEG classification is more difficult. Therefore, the reported results should be interpreted with this difference in mind.

DLob-based analysis provided additional explainable outputs. The generated DLob sentence had a complexity ratio of 64.90%. This result indicates a moderate level of structural regularity in the selected symbolic features. The TR symbol was the most dominant symbol in the DLob output, while frontal and parietal symbols were also observed. These findings show a temporal-dominant but distributed symbolic pattern. However, these outputs are model-based explanations. They should not be considered direct clinical connectivity results, seizure localization results, or anatomical ground truth.

The proposed model is lightweight and computationally simple when compared with many deep learning models. It does not require patch embedding, attention computation, or deep model training. Therefore, it may be suitable for standard computing environments and future embedded implementations. However, this practical use has not yet been tested in real-time or embedded systems. In addition, the possible use of OpT-centric XFE in non-invasive BCI applications remains a future research direction. It must be validated on task-specific, online, and real-time BCI datasets before any strong conclusion can be made.

The introduced OpT-centric XFE is a lightweight, interpretable, and effective feature engineering framework for EEG signal classification. The results support its potential for epilepsy detection. However, broader validation is still needed. Future studies should test the model on larger multi-center datasets, different EEG devices, additional biomedical signal modalities, and real-time clinical or BCI scenarios.

## Data Availability

This dataset is available in Kaggle and can be downloaded using https://www.kaggle.com/datasets/buraktaci/turkish-epilepsy URL.

## References

[CR1] Ahmad I, Zhu M, Li G, Javeed D, Kumar P, Chen S (2024) A secure and interpretable AI for smart healthcare system: A case study on epilepsy diagnosis using EEG signals. IEEE J Biomedical Health Inf 28(6):3236–3247 10.1109/JBHI.2024.3366341 38507373

[CR2] Asadi A, Wiesman AI, Wiest C, Baillet S, Tan H, Muthuraman M (2025) Electrophysiological approaches to informing therapeutic interventions with deep brain stimulation. npj Parkinson’s Disease 11(1):20 39833210 10.1038/s41531-024-00847-3 PMC11747345

[CR3] Bektas O, Kirik S, Tasci I, Hajiyeva R, Aydemir E, Dogan S, Tuncer T (2024) ChMinMaxPat: investigations on violence and stress detection using EEG signals. Diagnostics 14(23):2666 39682574 10.3390/diagnostics14232666 PMC11640744

[CR4] Bhattacharyya S, Khasnobish A, Chatterjee S, Konar A, Tibarewala D (2010) *Performance analysis of LDA, QDA and KNN algorithms in left-right limb movement classification from EEG data.* Paper presented at the 2010 International conference on systems in medicine and biology

[CR5] Brown BM, Boyne AM, Hassan AM, Allam AK, Cotton RJ, Haneef Z (2024) Computer vision for automated seizure detection and classification: A systematic review. Epilepsia 65(5):1176–1202 38426252 10.1111/epi.17926

[CR6] Cambay VY, Baig H, Aydemir A, Tuncer E, T., Dogan S (2024a) Minimum and Maximum Pattern-Based Self-Organized Feature Engineering: Fibromyalgia Detection Using Electrocardiogram Signals. Diagnostics 14(23):2708 39682616 10.3390/diagnostics14232708 PMC11639778

[CR7] Cambay VY, Tasci I, Tasci G, Hajiyeva R, Dogan S, Tuncer T (2024b) QuadTPat: Quadruple Transition Pattern-based explainable feature engineering model for stress detection using EEG signals. Sci Rep 14(1):27320 39516226 10.1038/s41598-024-78222-8 PMC11549367

[CR8] Cao X, Zheng S, Zhang J, Chen W, Du G (2025) A hybrid CNN-Bi-LSTM model with feature fusion for accurate epilepsy seizure detection. BMC Med Inf Decis Mak 25(1):6 10.1186/s12911-024-02845-0 PMC11706039 39762881

[CR9] Chollet F (2017) *Xception: Deep learning with depthwise separable convolutions.* Paper presented at the Proceedings of the IEEE conference on computer vision and pattern recognition

[CR10] Cresto N, Givalois L, Badaut J, Janvier A, Genin A, Audinat E, Marchi N (2024) Bursts of brain erosion: seizures and age-dependent neurological vulnerability. Trends Mol Med 10.1016/j.molmed.2024.11.003 PMC12536483 39665957

[CR11] Daniyal SM, Hussain SMT, Abbasi FL, Hussain D, Abbasi MM, Amjad U (2024) A hybrid deep learning model for precise epilepsy detection and seizure prediction. Spectr Eng Sci 2(3):62–77

[CR12] Denoeux T (2000) A neural network classifier based on Dempster-Shafer theory. IEEE Trans Syst Man Cybernetics-Part A: Syst Hum 30(2):131–150

[CR13] Dişli F, Gedikpınar M, Fırat H, Şengür A, Güldemir H, Koundal D (2025) Epilepsy Diagnosis from EEG Signals Using Continuous Wavelet Transform-Based Depthwise Convolutional Neural Network Model. Diagnostics 15(1):84 39795612 10.3390/diagnostics15010084 PMC11719495

[CR14] Edelman BJ, Zhang S, Schalk G, Brunner P, Müller-Putz G, Guan C, He B (2024) Non-invasive brain-computer interfaces: state of the art and trends. IEEE Rev Biomed Eng 18:26–49 10.1109/RBME.2024.3449790 PMC11861396 39186407

[CR15] Edoho M, Mooney C, Wei L (2024) AI-Based Electroencephalogram Analysis in Rodent Models of Epilepsy: A Systematic Review. Appl Sci 14(16):7398

[CR16] Elshewey AM, Alhussan AA, Khafaga DS, Elkenawy E-SM, Tarek Z (2024) EEG-based optimization of eye state classification using modified-BER metaheuristic algorithm. Sci Rep 14(1):24489 39424849 10.1038/s41598-024-74475-5 PMC11492230

[CR17] Gardy L, Curot J, Valton L, Berthier L, Barbeau EJ, Hurter C (2025) Detecting fast-ripples on both micro-and macro-electrodes in epilepsy: A wavelet-based CNN detector. J Neurosci Methods 415:110350 39675676 10.1016/j.jneumeth.2024.110350

[CR18] Gavnholt L, Gesche J, Cerulli Irelli E, Krøigård T, Mangaard SB, Di Bonaventura C, Beier CP (2024) Unsupervised clustering of a deeply phenotyped cohort of adults with idiopathic generalized epilepsy. *Epilepsia*10.1111/epi.18225 39724391

[CR19] Goldberger J, Hinton GE, Roweis S, Salakhutdinov RR (2004) Neighbourhood components analysis. Adv Neural Inf Process Syst 17:513–520

[CR20] Hothorn T, Lausen B (2003) Bagging tree classifiers for laser scanning images: a data-and simulation-based strategy. Artif Intell Med 27(1):65–79 12473392 10.1016/s0933-3657(02)00085-4

[CR21] Hu D, Wu K, Fang Y, Jiang T, Gao F, Cao J (2025) STMemAE: An instance-level based spatio-temporal memory autoencoder for unsupervised vision-based Seizure detection. IEEE Trans Emerg Top Comput Intell 9(5):3298–3310

[CR23] Jackson AF, Bolger DJ (2014) The neurophysiological bases of EEG and EEG measurement: A review for the rest of us. Psychophysiology 51(11):1061–1071 25039563 10.1111/psyp.12283

[CR24] Kiranyaz S, Ince T, Iosifidis A, Gabbouj M (2020) Operational neural networks. Neural Comput Appl 32(11):6645–6668

[CR25] Kononenko I (1994) *Estimating attributes: Analysis and extensions of RELIEF.* Paper presented at the European conference on machine learning

[CR26] Li C, Li H, Dong X, Zhong X, Cui H, Ji D, Zhou W (2025a) CNN-Informer: A hybrid deep learning model for seizure detection on long-term EEG. Neural Netw 181:106855 39488107 10.1016/j.neunet.2024.106855

[CR27] Li Z, Chen B, Zhu N, Li W, Liu T, Guo L, Yan Z (2025b) Epileptic Seizure Detection in SEEG Signals using a Signal Embedding Temporal-Spatial-Spectral Transformer Model. *IEEE Transactions on Instrumentation and Measurement*

[CR28] Liu H, Setiono R (1995) *Chi2: Feature selection and discretization of numeric attributes.* Paper presented at the Proceedings of 7th IEEE international conference on tools with artificial intelligence

[CR29] Liu Y, Xu C, Wen Z, Dong Y (2025) Trust EEG epileptic seizure detection via evidential multi-view learning. Inf Sci 694:121699

[CR30] Maillo J, Ramírez S, Triguero I, Herrera F (2017) kNN-IS: An Iterative Spark-based design of the k-Nearest Neighbors classifier for big data. Knowl Based Syst 117:3–15

[CR31] Manohara N, Ferrari A, Greenblatt A, Berardino A, Peixoto C, Duarte F, Kreuzer M (2024) Electroencephalogram monitoring during anesthesia and critical care: a guide for the clinician. J Clin Monit Comput, 1–34 10.1007/s10877-024-01250-2 39704777

[CR32] Mekruksavanich S, Phaphan W, Jitpattanakul A (2025) Epileptic seizure detection in EEG signals via an enhanced hybrid CNN with an integrated attention mechanism. Math Biosci Eng 22(1):73–105 39949163 10.3934/mbe.2025004

[CR33] Nafea MS, Ismail ZH (2024) IMPRESS: Informative mutual patch representation for EEG semi-supervised learning in Seizure type classification. IEEE Access 13:8951–8962

[CR34] Pellinen J, Foster EC, Wilmshurst JM, Zuberi SM, French J (2024) Improving epilepsy diagnosis across the lifespan: approaches and innovations. Lancet Neurol 23(5):511–521 38631767 10.1016/S1474-4422(24)00079-6

[CR35] Peng H, Long F, Ding C (2005) Feature selection based on mutual information criteria of max-dependency, max-relevance, and min-redundancy. IEEE Trans Pattern Anal Mach Intell 27(8):1226–1238 16119262 10.1109/TPAMI.2005.159

[CR36] Pidvalnyi I, Kostenko A, Sudakov O, Isaev D, Maximyuk O, Krishtal O, Ortmann S (2025) Classification of epileptic seizures by simple machine learning techniques: application to animals’ electroencephalography signals. IEEE Access 12:162251–162266

[CR37] Rashed-Al-Mahfuz M, Moni MA, Uddin S, Alyami SA, Summers MA, Eapen V (2021) A deep convolutional neural network method to detect seizures and characteristic frequencies using epileptic electroencephalogram (EEG) data. IEEE J translational Eng health Med 9:1–12 10.1109/JTEHM.2021.3050925 PMC7851059 33542859

[CR38] Safavian SR, Landgrebe D (1991) A survey of decision tree classifier methodology. IEEE Trans Syst man cybernetics 21(3):660–674

[CR39] Sandler M, Howard A, Zhu M, Zhmoginov A, Chen L-C (2018) *Mobilenetv2: Inverted residuals and linear bottlenecks.* Paper presented at the Proceedings of the IEEE conference on computer vision and pattern recognition

[CR41] Sharma R, Meena HK (2024) Emerging Trends in EEG Signal Processing: A Systematic Review. SN Comput Sci 5(4):1–14

[CR40] Sharma P, Gupta P, Gill AR, Kumar S, Kumar P, Singhal P, Khan S (2024) Current Paradigms in Understanding Neuron Fluctuations, Factors, Regulation, Pathophysiology of Epilepsy: Advancements in Diagnosis, Treatment and Management—An Update. Indian J Clin Biochem, 1–23. 10.1007/s12291-024-01281-1 10.1007/s12291-024-01281-1 PMC13396113 42500342

[CR42] Shawly T, Alsheikhy AA, Said Y, AbuEid AI, Alzahrani AA, Alshdadi AA, Ahmed HE (2025) MAFBN: Multi-Attention Forward and Backward network to predict epileptic seizure. Biomed Signal Process Control 104:107574

[CR43] Simonyan K, Zisserman A (2014) Very deep convolutional networks for large-scale image recognition. *arXiv preprint arXiv:1409.1556*

[CR44] Tasci B (2023) Turkish Epilepsy EEG Dataset, https://www.kaggle.com/datasets/buraktaci/turkish-epilepsy

[CR45] Tasci I, Tasci B, Barua PD, Dogan S, Tuncer T, Palmer EE, Acharya UR (2023) Epilepsy detection in 121 patient populations using hypercube pattern from EEG signals. Inform Fusion 96:252–268

[CR46] Tuncer T, Dogan S, Baygin M, Tasci I, Mungen B, Tasci B, Acharya U (2024) Directed Lobish-based explainable feature engineering model with TTPat and CWINCA for EEG artifact classification. Knowl Based Syst 305:112555

[CR47] Vapnik V (1998) The support vector method of function estimation. Nonlinear Modeling. Springer, pp 55–85

[CR48] Vaswani A (2017) Attention is all you need. *Advances in neural information processing systems*

[CR49] Verma A, Shrivastava A, Chaturvedi D (2025) Epilepsy detection system using CWT and deep CNN. Artificial Intelligence in Biomedical and Modern Healthcare Informatics. Elsevier, pp 211–222

[CR50] Zhang Y, Zhou X, Witt RM, Sabatini BL, Adjeroh D, Wong ST (2007) Dendritic spine detection using curvilinear structure detector and LDA classifier. NeuroImage 36(2):346–360 17448688 10.1016/j.neuroimage.2007.02.044

[CR51] Zhao W, Wang W-F, Patnaik LM, Zhang B-C, Weng S-J, Xiao S-X, Zhou H-F (2024) Residual and bidirectional LSTM for epileptic seizure detection. Front Comput Neurosci 18:1415967 38952709 10.3389/fncom.2024.1415967 PMC11215953

